# Evaluation of satisfaction on additional postpartum care – a comparative, multicentre study

**DOI:** 10.1186/s12884-025-08061-6

**Published:** 2025-09-08

**Authors:** Kajsa Sandberg Kedfors, Mattias Molin, Ingela Lindh

**Affiliations:** 1 Department of Obstetrics and Gynecology, Sahlgrenska Academy at Gothenburg University, Sahlgrenska University Hospital, Medicinaregatan 3, Gothenburg, SE- 413 45 Sweden; 2 Statistic Consulting Group, Stigbergsliden 5, Gothenburg, SE- 414 63 Sweden

**Keywords:** Breastfeeding, Maternal health, Satisfaction, Postpartum care, Postpartum period, Postpartum program

## Abstract

**Background:**

A growing body of knowledge is questioning the timing of postpartum care (PPC) and suggesting a structural change. The primary aim was to evaluate individuals’ satisfaction with additional PPC, and the secondary aim was to identify different needs postpartum.

**Methods:**

This comparative study was conducted in six maternity clinics in Gothenburg, Sweden 2019–2020. A total of 1159 eligible individuals were enrolled. An intervention model of care was evaluated, where the intervention group received two postpartum care compared to standard care, one visit (seven weeks postpartum) to their midwives. The participants answered questionnaires in connection to all postpartum care visits and at one-year postpartum a follow-up questionnaire was sent out.

**Results:**

The data from a total of 958 of 1159 participants (82.7%) were analysed: 447 of 554 (80.7%) in the intervention group vs 511 of 605 (84.4%) in the standard group. The participants in the intervention group reported satisfaction with the early, additional visit 413/444 (93%); where primiparous individuals reported a higher appreciation compared to parous individuals; 223/233 (95.7%) vs 190/211 (90.0%), *P* = 0.051. The three-weeks postpartum visit was perceived as timely, 434/441 (98.4%). Participants attending the clinic situated in a low socioeconomic status area were less likely to report a preference for additional visits. Nearly half of the participants in the standard group 201/466 (43%) expressed a preference towards an additional and early visit and this was more common among primiparous, 127/254 (50%) vs parous 74/212 (34.9%) *P* = 0.001. Those in the standard group who expressed a need for earlier support reported a wish for discussing abdominal/vaginal problems and experience of giving birth. Primiparous 73/127 (57.7%) were more likely than parous individuals 28/74 (37.8%) *P* = 0.011 to express a need for additional support with breastfeeding.

**Conclusions:**

The results demonstrate an increased need for early and additional visits among primiparous, and important topics were abdominal/vaginal problems, experience of giving birth and breastfeeding. Information for individuals in low socioeconomic status areas needs to be expanded.

**Supplementary Information:**

The online version contains supplementary material available at 10.1186/s12884-025-08061-6.

## Background

Readily available and quality care is a prerequisite for people’s well-being postpartum. Historically, maternal care during the postpartum period has in many countries, e.g. United Kingdom and in the United States of America been treated as a single event with one visit scheduled approximately six weeks postpartum [1]. This has recently been reviewed in both English (NICE recommendations) as well as American College of Obstetrician and Gynecologists (ACOG) where ACOG now recommends postpartum care to be an ongoing process, rather than a single encounter, with services and support tailored to each postpartum individuals needs [1–3]. The World Health Organization (WHO) recommendations regarding postpartum care recommends a continuum of increased postpartum care focuses not only on survival but also on the quality of care and emphasizes additional support to vulnerable individuals [2, 4]. In Sweden during the last decades the inpatient stay after childbirth has undergone major changes. It has been shortened from a median of six days stay during the 70 s, up to todays recommended inpatient time for healthy mothers and infants to be approximately six to eight hours after giving birth [5, 6]. In Sweden, routine postpartum care after hospital discharge includes a single follow-up visit between 6 and 16 weeks postpartum. This visit takes place at the same maternity clinic where the individual received prenatal care and is conducted by the midwife who provided their antenatal care. A gynaecologist will only see the postpartum individuals if there are complications during pregnancy or childbirth [7]. In the last Swedish national evaluation regarding perinatal care, the highlighted and most urgent area for improvement was the postpartum care [8]. The problems noted in this national report were among others absence of early postpartum care after discharge from hospital e.g. problems concerning reproductive issues caused by birth and breastfeeding. The postpartum care visit (PPCV) at seven weeks was perceived as late. This has also been reported in an English study where primiparous described problems in navigating in the health care system during the postpartum period [9].

Postpartum care is supposed to meet all the different needs that can arise after childbirth and ought to address numerous possible issues such as physiological changes, perform physical examinations, offer breastfeeding support, present an opportunity to discuss the experience of giving birth, assess psychological status, provide sexual health advice as well as contraceptive counselling [7, 10].

A recently conducted overview of the postpartum period concluded that individuals are at risk for different potential difficulties with varying severity levels, such as infection, pain, depression and breastfeeding issues [11].

A scoping review by Benova et al. concluded that there were challenges developing and implementing routine postpartum guidelines. The authors pointed out the lack of evidence on how processes of individualizing postpartum care provision can be applied in practice to support health workers in providing person-centred care in various global settings [12].

The primary aim of this study was to investigate the hypothesis that earlier and repeated PPCVscompared to one standard PPCV would increase individuals’ satisfaction, and the secondary aim was to identify individuals’ different postpartum needs.

## Methods

This is a two-arm, comparative multicentre study which took place in six different maternity clinics in Gothenburg and Molndal, Sweden. The relevant stakeholders for this study were postpartum individuals and midwifes working in these clinics. The selected clinics were together representative of the population in Gothenburg and surroundings, representing geographical areas with a range in socioeconomic status (SES), (Angered, Gibraltar, Linné, Molndal, Gamlestaden, Frolunda) [13]. The first part of the main study including the full methodology has been published elsewhere [14]. The study was conducted between January 11, 2019, to June 1, 2020. The one-year follow up was conducted from March 11, 2020 to June 15, 2021. In preparation for the study, several pregnant individuals were involved in reading and evaluating the written informed consent and the questionnaires. Eligible participants were pregnant individuals attending antenatal care at one of the included maternity clinics during the recruitment period in pregnancy week 37, aged 18-years or older and fluent in Swedish or English. The eligible individuals were approached and informed about the study at their visit in pregnancy week 37, either by a clinic nurse or a midwife, depending on the clinic. If the individual accepted to be a participant in the study, they signed the consent. Participants were not informed regarding the allocation procedure. The allocation was systematically based on date of birth: individuals born on even-numbered dates were assigned to the intervention group, while those born on odd-numbered dates were assigned to the standard care group. Group allocation was conducted by midwives at each study centre. No form of compensation was given to the participants. The week 37 prenatal visit is a standard practice for both primiparous and parous pregnant individuals. This timing was chosen to minimize the risk of scheduling complications due to premature births, which occur in approximately 5% of deliveries in Sweden. For all participants irrespective of standard or intervention group, immediate postpartum care was hospital based. The scope of this study is postpartum care given in the outpatient setting that took place in six different maternity clinics as described. The study compares standard postpartum care after discharge from hospital with an intervention model of care. Standard postpartum care comprised of one PPCV (approximately seven weeks postpartum) at the maternity clinic provided by midwives (no home visits since this weren’t routine praxis in Sweden). At the visit individuals could discuss the experience of giving birth, breastfeeding, problems concerning reproductive issues caused by birth, contraceptive counselling, and undergo a physical examination. The intervention group was given an early and additional PPCV (a first visit three weeks after birth) followed by the seven weeks postpartum visit. The early visit followed a clear structure to identify any problems that frequently arise during the first weeks following childbirth (vaginal/abdominal problems, opportunity to discuss experience of giving birth, breastfeeding, contraception and other acute issues). The choice of timing for the early additional PPCV was due to a previously identified shortage in support, described by postpartum individuals in a national survey [5]. The second and later visit at seven weeks was aimed to address follow up topics from the earlier three week visit and to offer a physical examination. At each visit, the participants were cared for by their regular midwife. In connection to their different visits, all participants received questionnaires via a Quick Response-code, by their regular provider (midwife). One year postpartum, all participants in the study were sent a follow-up questionnaire (no physical visit) to their smartphone. Three reminders were sent out by a short message service. We collected the outcome data from the questionnaires using esMaker [15], a web-based tool for surveys. The questionnaires were designed with a mix of answer options, five-point scales or free text answers depending on the question. Results related to initiation and method of contraceptive use are presented in an earlier paper from the same comparative multicentre study [14]. The Transparent Reporting of Evaluations with Nonrandomized Designs (TREND) guidelines were used to ensure thorough and transparent reporting [16]. Our outcome measures were preference for and satisfaction with two PPCVs compared to one PPCV. The outcome measures at the one-year follow-up were to identify the most important topics discussed at the PPCVs.

### Statistical analyses

Categorical data are presented as numbers and percentages and continuous data are presented with means and standard deviations, and where appropriate with median, minimum, maximum and number of observations. Difference in ordered categorical data between two groups were tested using Mantel–Haenszel Chi Square tests and Fishers exact tests for dichotomous data with differences in percentage and 95% confidence interval (CI). Chi 2 test was performed for unordered categorical variables and t-test for continuous variables. Subgroup analyses were performed by age, parity, method of birth, country of birth and maternity clinics. Interaction analysis was performed for maternity clinics and groups regarding satisfaction with care, using logistic regression analysis. Statistical analysis was performed using SAS 9.4, by SAS Institute Inc., Cary, NC, USA, following a predetermined statistical analysis plan. All tests were performed as two-sided with alpha 0.05. A calculated sample size of a minimum of 400 in each group was required to achieve 80% power at a significance alpha level at 0.05 and to detect a 10% difference in dichotomous outcomes between the groups (intervention 50% and standard 40% groups).

## Results

Between January 11, 2019, and June 1, 2020, we screened pregnant people at pregnancy week 37 at six maternity clinics in Sweden. A total of 1626 individuals were approached (presented as a flowchart in earlier published paper [14]) and out of these, 467 of 1626 (28.7%) were excluded due to not meeting the inclusion criteria. We enrolled a total of 1159 eligible individuals in the study, 554 of 1159 (47.8%) in the intervention group and 605 of 1159 (52.2%) in the standard group [14]. Data from a total of 958 of 1159 participants (82.7%) were analysed: 447/511 (80.7%) in the intervention group and 511/605 (84.5%) in the standard care group. Among those lost to follow-up, a higher proportion were foreign-born 188/201 (94%) compared to 12/201 (6%) who were born in Sweden.

For the one-year follow-up, conducted from March 11, 2020, to June 15, 2021, a total of 958 participants were sent a Link to a questionnaire. A total of 806 of 958 (84.1%) participants completed the questionnaire, with response rates of 382 of 447 (85.5%) in the intervention group and 424 of 511 (82.9%) in the standard care group [14].

Background demographics were similar between the intervention and standard groups (Table 1). More than 90% reported cohabitating and 20% were foreign-born (Table 1).

**Table 1:** Baseline data (*n* = 958)

Variable	Intervention (*n* = 447)	Standard (*n* = 511)	*p*-value
Age (years)			
Mean (SD)	31.6 (4.4)	31.7 (4.4)	0.75
Age primiparous (years)			
Mean (SD)	30.6 (4.5)	30.5 (3.9)	0.90
Age parous (years)			
Mean (SD)	32.7 (4.1)	33.0 (4.4)	0.40
Parity	n (%)	n (%)	
Primiparous	235 (52.6%)	278 (54.4%)	
Parous	212 (47.4%)	233 (45.6%)	0.62
Birth mode	n (%)	n (%)	
Vaginal	353 (79.0%)	418 (81.8%)	0.31
Vacuum extraction	21 (4.7%)	25 (4.9%)	
Caesarean section	73 (16.4%)	68 (13.3%)	
Relationship status	n (%)	n (%)	
Cohabitating	421 (94.4%)	475 (93.3%)	
Single	10 (2.2%)	9 (1.8%)	
Live-apart	15 (3.4%)	25 (4.9%)	0.44
Country of birth	n (%)	n (%)	
Sweden born	328 (73.4%)	405 (79.3%)	0.039
Foreign born	119 (26.6%)	106 (20.7%)	
BMI 37th pregnancy week	n (%)	n (%)	
Normal (18.5–25)	78 (17.7%)	82 (16.2%)	0.48
Overweight (25–30)	201 (45.7%)	231 (45.6%)	
Obese (> 30)	161 (36.6%)	194 (38.3%)	

Continuous variables are presented with median and standard deviation (SD)

Categorical variables are presented with numbers and percentage, *n* (%). *n* = numbers

*BMI* Body Mass Index (kg/m2)

One maternity clinic situated in the area with the lowest SES population (Angered) was the clinic that deviated most from the other five clinics with a higher proportion of foreign-born participants and more participants not cohabiting with the other parent. Background demographic data divided by clinics is described in more detail (Supplementary Table 1).

**Fig. 1:**
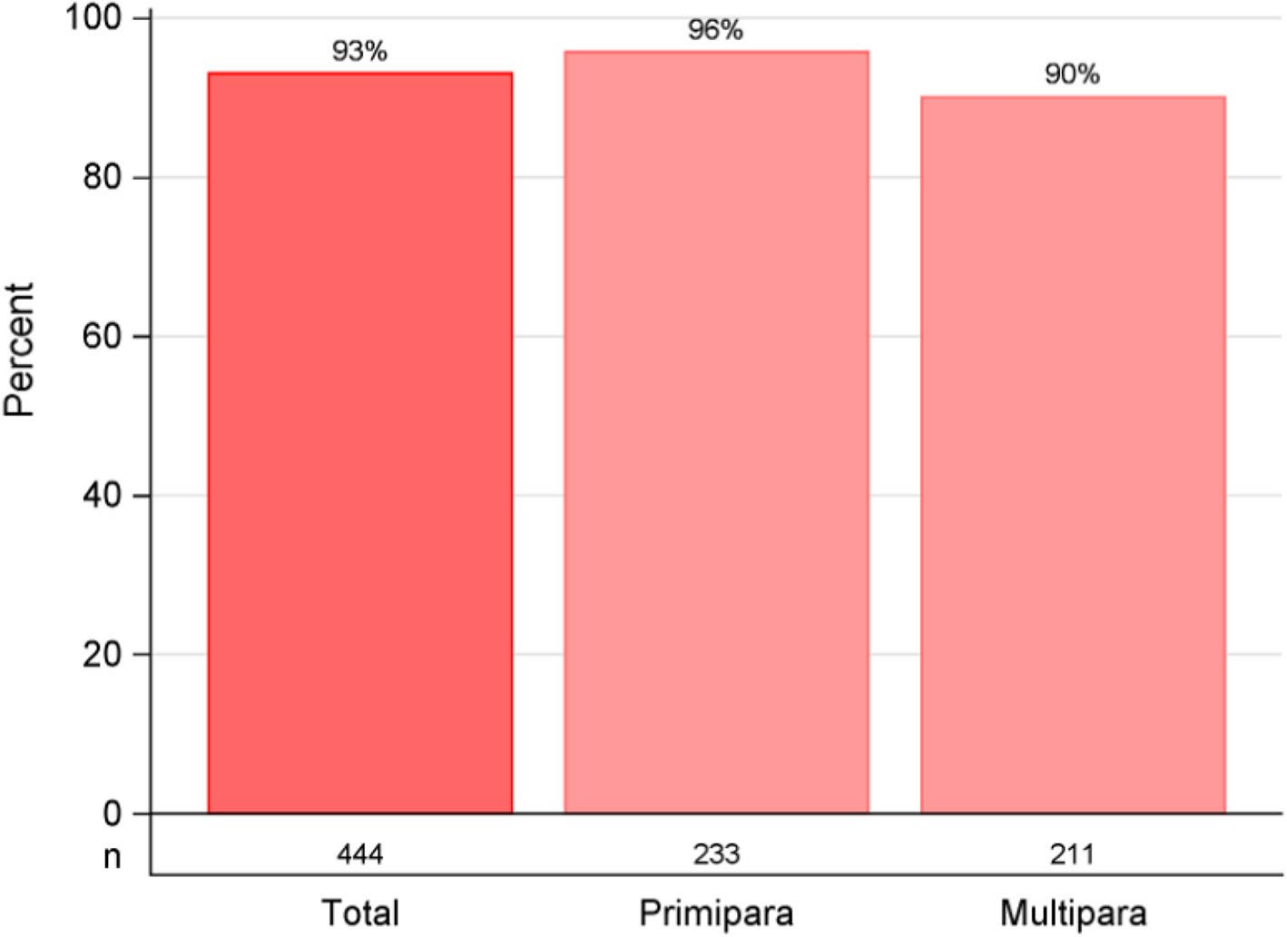
Satisfied with two visits, intervention group (visits at three and seven weeks postpartum)

**Fig. 2:**
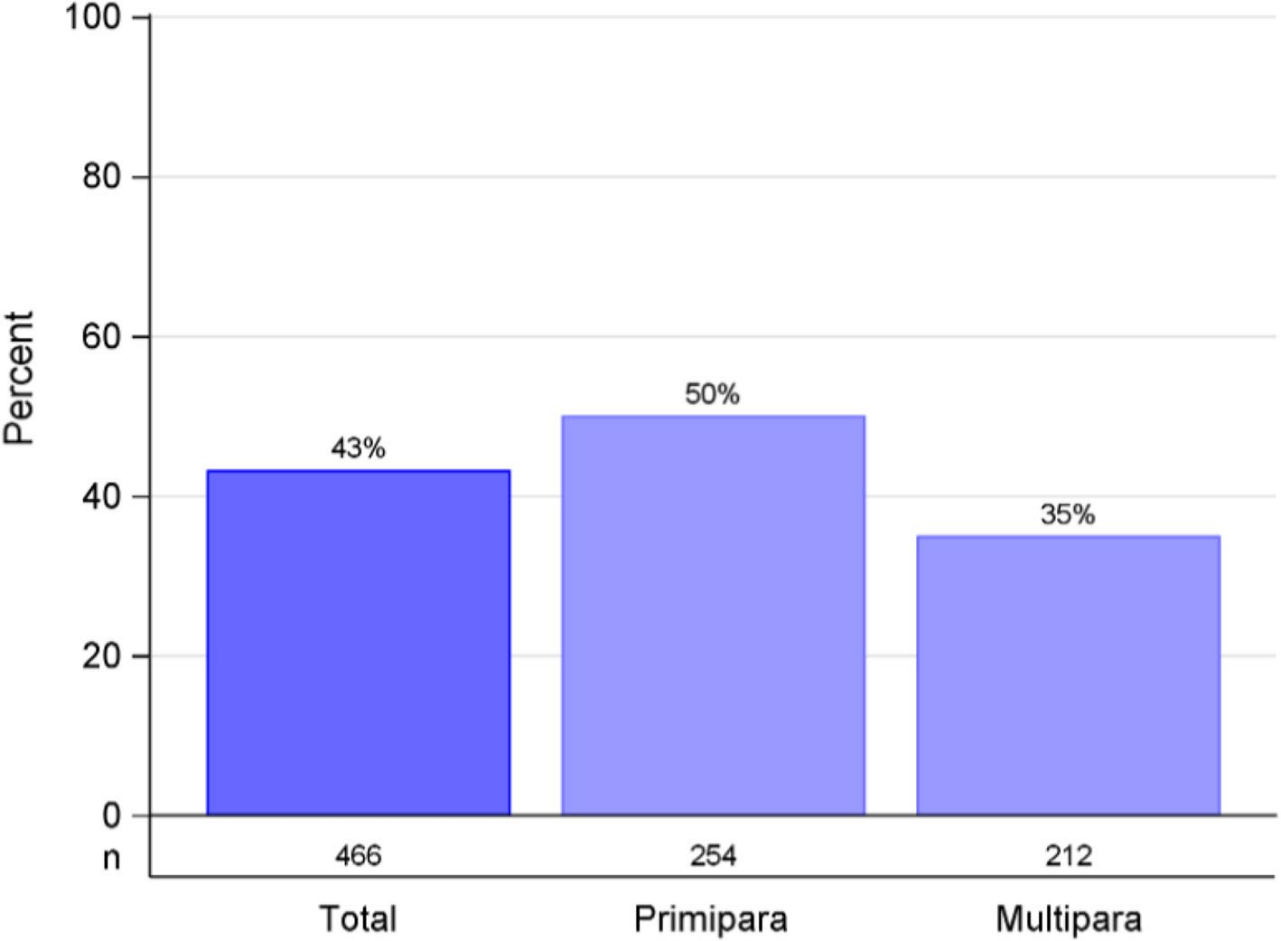
Preference to receive two visits, standard group (one visit at seven weeks postpartum)

**Fig. 3:**
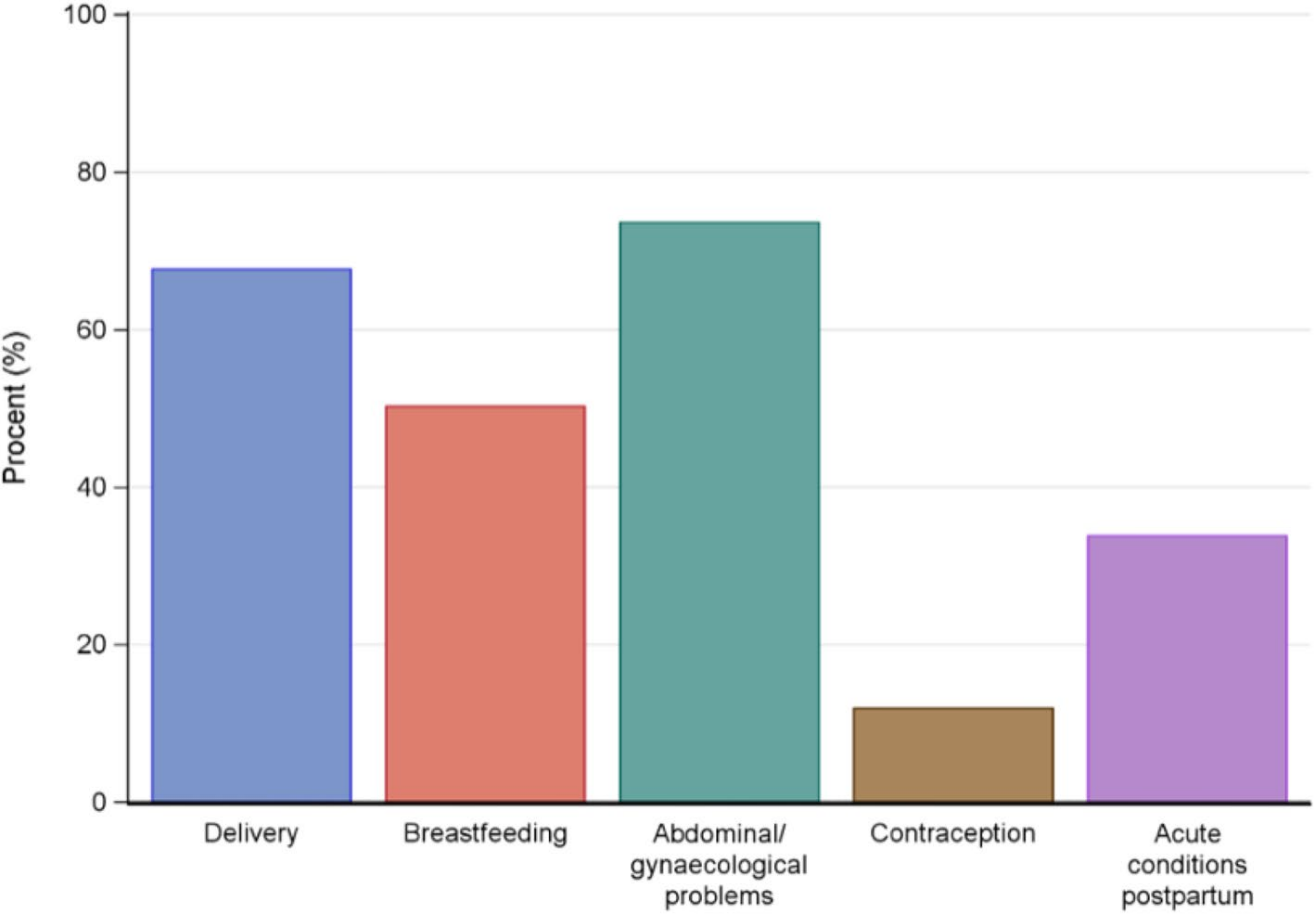
Prioritized topics expressed from the standard group if an early PPCV had been offered (*n* = 201)

Participants in the intervention group 413/444 (93%) expressed high satisfaction with receiving early and additional PPCVs; primiparous individuals reported a higher appreciation compared to parous individuals: 223/233 (95.7%) vs 190/211 (90.0%) *P* = 0.051 (Fig. 1). Of the participants in the standard group (one visit), less than half, 201/466 (43%) expressed a preference for receiving two visits (three and seven weeks postpartum) if they had the possibility to choose an early and additional PPCVs (Fig. 2). Among these 43% a difference by parity was seen: primiparous, 127/254 (50%) vs parous individuals, 74/212 (34.9%), a difference of 15.1 (95% CI 5.8—24.4) *P* = 0.001 (Fig. 2). An interaction analysis for maternity clinics regarding preference for one or two PPCVs between the groups showed a difference in total of 36% in favour for two visits (*P* interaction = 0.095) except at one of the six maternity clinics, situated in the low SES area, where no difference regarding preference for one or two visit was found. When participants in the intervention group evaluated their experience of the early visit (three weeks postpartum) they reported it as timely, 434/441 (98.4%) only 7/441 (1.6%) reported that the visit was too soon after childbirth. Most of the individuals in the intervention group, 425/441 (96.4%) stated that they preferred physical visits rather than a phone call 12/441 (2.7%) or videocall 4/441(0.9%). When asking the participants in the standard group what they had preferred to discuss if they had been offered an earlier and additional visit closer to their birth, the most important topics identified were: support regarding abdominal and/or vaginal problems, experience of giving birth and breastfeeding (Fig. 3). There was a difference when comparing primiparous to parous people regarding different topics, where support with breastfeeding was the most prioritized topic among primiparous, 73/127 (57.5%) compared to parous individuals 28/74 (37.8%), a difference of 19.7 (95% CI 4.6—34.7) *P* = 0.011.

No difference in breastfeeding rates between the two groups was reported at seven weeks postpartum: intervention group 390/447 (87.3%) and standard group 450/511 (88.1%).

**Fig. 4:**
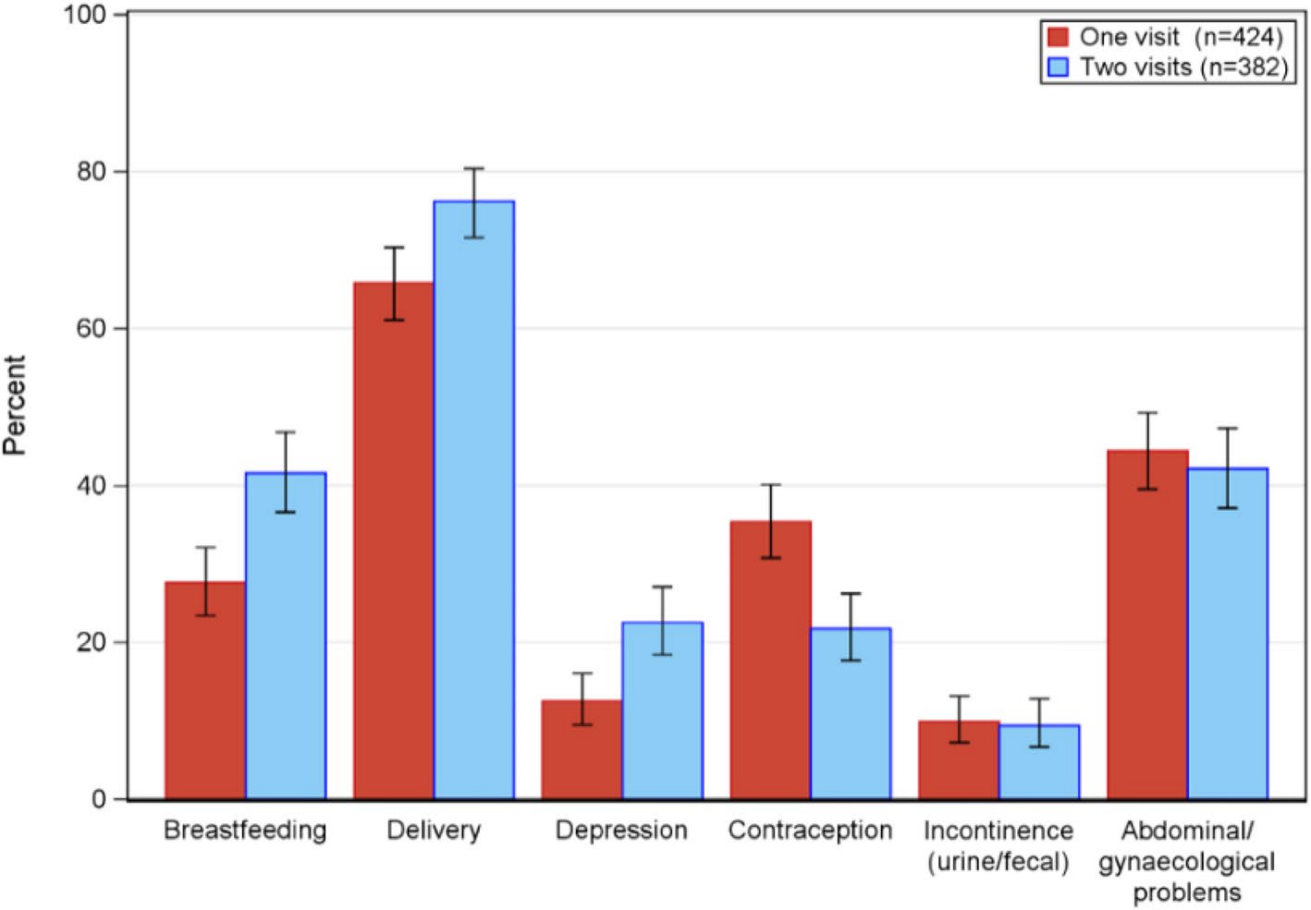
Most prioritized topics to discuss postpartum based on one-year follow up survey (*n* = 806)

At the one-year follow up, all participants had the opportunity to give feedback and evaluate the most important topics discussed at their respective PPCV. The secondary outcome shows that the topics of breastfeeding, delivery and abdominal/vaginal problems were reported as the top prioritized (Fig. 4). Breastfeeding was a more important topic in the intervention group at their three-week visit compared to the one and only seven-week visit in the standard group 159/382 (41.6%) and 117/424 (27.6%) respectively, a difference of 14.0 (95% CI 7.3—20.8) *P* = 0.011. Similar results were seen for the need to discuss the experience of giving birth: intervention group 291/382 (76.2%) vs standard group 279/424 (65.8%), a difference of 10.4 (95% CI 3.9—16.8) *P* = 0.002. At the one-year follow up, the participants in the intervention group (at their three-week visit) expressed the topic of depression as a more important issue to discuss 86/382 (22.5%) compared to the participants belonging to the standard care group at the one and only seven-week visit 53/424 (12.5%), a difference of 10.0 (95% CI 4.5—15.5) *P* = 0.002 (Fig. 4). Among the standard group, 32/410 (7.8%) reported an ongoing pregnancy at the one-year follow up, compared to 15/369 (4.1%) in the intervention group, a difference of 3.7 (95% CI 0.2—7.3) *P* = 0.040.

## Discussion

Our results have shown contentment with an early and additional PPCV, and this is in line with previous knowledge suggesting that one PPCV after childbirth might be inadequate to respond to the individuals postpartum needs [2]. There was high satisfaction among the majority of participants in the intervention group who received an early and additional PPCV, where primiparous individuals reported the highest appreciation. However, less preference for additional visits was reported from individuals in the clinic situated in the low SES area. Among the individuals in the standard care group, half of all primiparous individuals expressed a need for earlier postpartum care. This group expressed that if an early and additional PPCV had been available, the topics of importance to discuss at three-weeks postpartum were the experience of giving birth, abdominal/vaginal problems, and breastfeeding.

The WHO outlines goals for postpartum care aimed to create a positive experience and support individuals and their babies in achieving their full potential for health and well-being [17]. This implies not only survival for the parturient and neonate, but it also indicates a measure of quality of care and satisfaction for the individual. Previously, the focus has been mainly on coverage and access to postpartum care. An inventory of the content of postpartum care to ensure that the existing care is of sufficiently good quality has been proposed [18].

In the last Swedish national evaluation regarding perinatal care, postpartum care was identified as in need of improvement [8]. People often report a feeling of insufficient support in the period from childbirth and the first PPCV [19]. A cohort study assessing postpartum individuals unmet needs six to eight weeks postpartum concluded that many had problems with episiotomy, physical changes, and other physical complications [20]. This is in line with our results since study participants in the standard group described a need for early support regarding abdominal/gynaecology issues prior to the seven week visit. In our study, primiparous individuals reported the highest satisfaction, and a need for additional PPCVs. Primiparous individuals may require additional support to feel secure and confident in their recovering from, and in their parenting role [21]. A scoping review examining factors associated with maternal parental self-efficacy concluded that self-efficacy tends to increase with the number of children. In that study, primiparous mothers reported lower levels of maternal parental self-efficacy [22]. The participants in our study with a preference for the additional and earlier visit reported that discussing the experience of giving birth at seven weeks postpartum was too late, suggesting a need for early and individual support. This is in line with a Danish study describing how peoples’ perception of giving birth changed from one week postpartum to six weeks postpartum. The perception of the birth experience seemed to shift towards a more negative experience at six weeks postpartum [23].

The results show some differences regarding preference for additional postpartum care, where individuals attending the clinic in the lowest SES area with the distinguished as the only included clinic with no reported need of additional care. Maternal health literacy has been shown to be lower among individuals with low SES [24]. Furthermore, in Sweden, attendance in postpartum care is associated with sociodemographic factors where lower participation rate is seen among less educated and migrant individuals [25]. This inequity has been described in an English study as well, in which individuals living in deprived areas were less likely to attend PPCV [26]. Likewise, an U.S. study described an association between reduced levels of attendance in postpartum care and sociodemographic factors [27]. The Inverse Care Law posits that those with the greatest need for healthcare often receive the least or most inadequate care [28]. This principle emphasizes that individuals facing social disadvantage are less likely to access high-quality healthcare compared to those with fewer needs and higher SES [28]. Even in countries where healthcare is subsidized and theoretically available on equal terms to all, disparities in utilization persist. Smith et al. describe maternal health agency as the capacity to form preferences. Lower levels of health agency are often associated with lower SES [29]. This may help explain why individuals living in areas with the lowest SES were expressed no preference for additional postpartum care.

Our results show that two visits were not enough to increase breastfeeding rates at seven weeks postpartum. We believe that even earlier identification of breastfeedingproblems and repeated breastfeeding support is needed. According with previous research, Gianni et al. reported that 63% of postpartum people experienced difficulties with breastfeeding during the first four weeks following childbirth [30].

Our study found that more than one fifth of participants expressed depressive symptoms at the early visit, three weeks after birth. A previous systematic review suggested a need for early support on postpartum depression since presence of depression creates unfavourable conditions for both the parturient and the neonates [31]. Among participants in the standard group at seven weeks, one in ten reported depressive symptoms, which is comparable to international population figures for postpartum depression [32].

The strengths of this study include its design within a real-world setting, a large sample size, and high response rates near the time of delivery and at the one-year follow-up. The analysed population is comparable to the national average regarding the mean age of primiparous and parous individuals though it may not be generalizable to other settings. Limitations in this study were that blinding of participants was not possible and the pregnant individuals were not randomly assigned to the intervention and the standard group. However, participants were not informed of the allocation method and therefore could not choose to participate based on any prior expectations regarding group assignment. One more limitation was that only individuals fluent in Swedish or English were included, as it was not feasible to use interpreters for translating questionnaires, which may affect the generalizability. Analysing baseline characteristics of participants lost to follow-up at seven weeks compared to those who completed the study revealed a higher number of foreign-born participants in the lost to follow-up subset.

## Conclusion

In conclusion, the results suggest that postpartum care ought to be differentiated and tailored to fit the needs of the individual. Our study supports early and additional postpartum care, particularly for primiparous individuals, who displayed greater needs. These findings are important for healthcare providers as well for decision makers when reviewing postpartum care guidelines. The delivery of postpartum care should be further explored to increase accessibility to care in low SES areas, to ensure support for breastfeeding and, to offer early follow-up regarding depression.

## Supplementary Information

Supplementary file 1: Supplementary Table 1. Baseline divided by clinic (*n* = 958).

## Data Availability

The data that support the findings of this study are available from the corresponding author, KSK, upon reasonable request.

## Abbreviations

CI: Confidence interval
CS: Caesarean section
PPCV: Postpartum care visit
SES: Socioeconomic status
TREND: The Transparent Reporting of Evaluations with Nonrandomized Designs
VE: Vacuum Extraction
WHO: The World Health Organization
