# Supplementary material for: Evaluation of satisfaction on additional postpartum care – a comparative, multicentre study
Source: BMC Pregnancy Childbirth. 2025 Sep 8;25:930. doi: 10.1186/s12884-025-08061-6 (PMC12418634; doi:10.1186/s12884-025-08061-6)
Supplement: Supplementary file 1 — Supplementary Table 1. Baseline divided by clinic (n = 958). [file 12884_2025_8061_MOESM1_ESM.docx]

Supplementary Table 1. Baseline divided by clinic (n=958)

| Variable | Total (n=958) | Angered (n=62) | Gibraltar (n=153) | Linné (n=139) | Mölndal (n=160) | Gamlestaden (n=154) | Frölunda (n=290) |
| --- | --- | --- | --- | --- | --- | --- | --- |
| Age (years) | | | | | | | |
| Mean (SD) | 31.6 (4.4) | 30.3 (4.5) | 32.0 (3.9) | 32.5 (4.2) | 31.5 (4.6) | 31.5 (4.4) | 31.5 (4.5) |
| Age primiparous (years) | | | | | | | |
| Mean (SD) | 30.6 (4.2) | 28.6 (3.7) | 31.2 (3.9) | 31.8 (4.1) | 29.8 (4.4) | 30.5 (4.3) | 29.8 (4.1) |
| Age parous (years) | | | | | | | |
| Mean (SD) | 32.9 (4.3) | 30.8 (4.7) | 33.8 (3.4) | 34.2 (4.2) | 33.2 (4.1) | 32.4 (4.2) | 32.9 (4.3) |
| Parity | *n* (%) | *n* (%) | *n* (%) | *n* (%) | *n* (%) | *n* (%) | *n* (%) |
| Primiparous | 513 (53.5%) | 16 (25.8%) | 108 (70.6%) | 100 (71.9%) | 82 (51.3%) | 73 (47.4%) | 134 (46.2%) |
| Parous | 445 (46.5%) | 46 (74.2%) | 45 (29.4%) | 39 (28.1%) | 78 (48.8%) | 81 (52.6%) | 156 (53.8%) |
| Birth mode | *n* (%) | *n* (%) | *n* (%) | *n* (%) | *n* (%) | *n* (%) | *n* (%) |
| Vaginal | 771 (80.5%) | 56 (90.3%) | 120 (78.4%) | 97 (69.8%) | 128 (80.0%) | 129 (83.8%) | 241 (83.1%) |
| Vacuum extraction | 46 (4.8%) | 2 (3.2%) | 12 (7.8%) | 11 (7.9%) | 8 (5.0%) | 1 (0.6%) | 12 (4.1%) |
| Caesarean sectio | 141 (14.7%) | 4 (6.4%) | 21 (13.7%) | 31 (22.3%) | 24 (15.0%) | 24 (15.5%) | 37 (12.8%) |
| Relationship status | *n* (%) | *n* (%) | *n* (%) | *n* (%) | *n* (%) | *n* (%) | *n* (%) |
| Cohabitating | 896 (93.8%) | 49 (79.0%) | 148 (98.7%) | 132 (95.0%) | 157 (98.1%) | 139 (90.3%) | 271 (93.4%) |
| Single | 19 (2.0%) | 3 (4.8%) | 1 (0.7%) | 4 (2.9%) | 1 (0.6%) | 4 (2.6%) | 6 (2.1%) |
| Live-apart | 40 (4.2%) | 10 (16.1%) | 1 (0.7%) | 3 (2.2%) | 2 (1.3%) | 11 (7.1%) | 13 (4.5%) |
| Country of birth | *n* (%) | *n* (%) | *n* (%) | *n* (%) | *n* (%) | *n* (%) | *n* (%) |
| Sweden born | 733 (76.5%) | 25 (40.3%) | 127 (83.0%) | 128 (92.1%) | 129 (80.6%) | 101 (65.6%) | 223 (76.9%) |
| Foreign born | 225 (23.5%) | 37 (59.7%) | 26 (17.0%) | 11 (7.9%) | 31 (19.4%) | 53 (34.4%) | 67 (23.1%) |
| BMI 37^th^ pregnancy week | *n* (%) | *n* (%) | *n* (%) | *n* (%) | n (%) | *n* (%) | *n* (%) |
| Normal (18.5-25) | 160 (16.9%) | 6 (9.7%) | 31 (20.8%) | 32 (23.0%) | 24 (15.3%) | 30 (19.6%) | 37 (12.9%) |
| Overweight (25-30) | 432 (45.6%) | 17 (27.4%) | 82 (55.0%) | 69 (49.6%) | 68 (43.3%) | 63 (41.2%) | 133 (46.3%) |
| Obese (>30) | 355 (37.5%) | 39 (62.9%) | 36 (24.2%) | 38 (27.3%) | 65 (41.4%) | 60 (39.2%) | 117 (40.8%) |

Continuous variables are presented with median and standard deviation (SD).

Categorical variables are presented with numbers and percentage, *n* (%). n=numbers

BMI – Body Mass Index (kg/m2)
